# Rapid optimization of gene dosage in *E. coli *using DIAL strains

**DOI:** 10.1186/1754-1611-5-10

**Published:** 2011-07-25

**Authors:** Joshua T Kittleson, Sherine Cheung, J Christopher Anderson

**Affiliations:** 1 Department of Bioengineering, University of California, Berkeley, USA; 2 Department of Bioengineering, University of California, San Diego, USA; 3 Berkeley National Laboratory, Physical Biosciences Division; QB3: California Institute for Quantitative Biological Research; 327 Stanley Hall, Berkeley, CA 94720, USA

## Abstract

**Background:**

Engineers frequently vary design parameters to optimize the behaviour of a system. However, synthetic biologists lack the tools to rapidly explore a critical design parameter, gene expression level, and have no means of systematically varying the dosage of an entire genetic circuit. As a step toward overcoming this shortfall, we have developed a technology that enables the same plasmid to be maintained at different copy numbers in a set of closely related cells. This provides a rapid method for exploring gene or cassette dosage effects.

**Results:**

We engineered two sets of strains to constitutively provide a *trans*-acting replication factor, either Pi of the R6K plasmid or RepA of the ColE2 plasmid, at different doses. Each DIAL (different allele) strain supports the replication of a corresponding plasmid at a constant level between 1 and 250 copies per cell. The plasmids exhibit cell-to-cell variability comparable to other popular replicons, but with improved stability. Since the origins are orthogonal, both replication factors can be incorporated into the same cell. We demonstrate the utility of these strains by rapidly assessing the optimal expression level of a model biosynthetic pathway for violecein.

**Conclusions:**

The DIAL strains can rapidly optimize single gene expression levels, help balance expression of functionally coupled genetic elements, improve investigation of gene and circuit dosage effects, and enable faster development of metabolic pathways.

## Background

Optimizing desired outcomes by varying a design parameter is a staple of almost every engineering field, from mechanical engineers tweaking blade angles on a wind turbine to civil engineers altering the timing of traffic lights. Similarly, genetic engineers alter gene expression levels to optimize some desirable phenotype. Strong overproduction of single proteins can impose a metabolic burden on *E. coli*, and often a lower expression level leads to improved phenotype [1]. In multi-subunit proteins and genetic circuits, expression of particular proteins often needs to be balanced for proper function (e.g. [2,3], and [4]). Extensive work has established methods for achieving expression of a gene or operon at a particular level, including control of transcription using standard promoter sets [5], modulation of RNA processing [6], and control of translation through ribosome binding site (RBS) manipulation [7]. However, using these tools to investigate the desired expression level of a single gene or operon requires cloning for each level to be tested. Using inducible promoter systems to probe multiple expression levels can rapidly determine an approximate desired expression level, but does not provide a genetically encoded solution, which can be useful for downstream applications. For optimizing multi-operon constructs, fewer tools exist. Generating large numbers of repeats in the genome is labor intensive [8], while strategies that increase plasmid copy number upon induction provide a narrow range of copy numbers [9], cause runaway replication [10,11], or are incompatible with constructs using the common P_BAD _promoter [12]. To address these shortcomings and allow researchers to explore the effect of copy number on genetic devices, we have exploited an underutilized control point: plasmid copy number.

Genetic engineers working in *E. coli *are blessed with a wide range of plasmid systems and plasmid copy numbers to choose from, ranging from single copy BACs to ~500 copy pUC plasmids [13]. However, to take advantage of copy number differences, each gene or device of interest has to be cloned into different plasmids, necessarily changing the local genetic context along with the copy number. A notable exception to this rule is the gamma origin of the R6K plasmid, which requires the *trans*-acting Pi protein to initiate replication [14]. Different alleles of *pir *integrated into the *E. coli *genome are known to support R6K plasmids at different copy numbers (e.g. *pir+ *and *pir116 *[15]), meaning that the genetic context on the plasmid itself remains unaltered. Similarly, the orthogonal replicon of ColE2-P9 also uses a *trans*-acting factor, RepA, to support the origin of replication [16].

In this work, we generate two sets of strains bearing different alleles (DIAL) of *pir *or *repA *to support the same plasmid at a wide range of copy numbers. We then characterize the copy number, cell-to-cell variability, and stability of plasmids in both sets of DIAL strains. We illustrate their utility on a model system by examining expression of the violacein biosynthesis pathway [17] at different copy numbers. The results demonstrate that artificially re-regulating replication factor expression from the genome can produce stable plasmid copy numbers, that phenotype varies with copy number, and that DIAL strains can accelerate development of genetic devices.

## Results and Discussion

### ColE2 as a *Trans*-Activated Origin

A previous report [18] identified a minimal 32 bp region of ColE2 sufficient to support replication of a plasmid when *repA *is provided in *trans*. We first recapitulated this behaviour by transforming a plasmid bearing the ColE2 minimal origin (pBjk2164-jtk2619) into cells constitutively expressing the *trans*-acting *repA *gene from a plasmid (MC1061 + pBca9145-jtk2627). Although we observed transformants, they exhibited small colony morphologies. Presuming this observation to reflect plasmid instability (as suggested by data in [19]), we repeated the experiment using a larger 470 bp fragment of the ColE2 plasmid as the origin (pBjk2164-jtk2642) in the hope that any context dependent influence on the minimal origin would be eliminated, or that non-essential factors contributing to robustness would be captured. This yielded colonies with morphologies indistinguishable from untransformed cells (data not shown).

### Orthogonality of R6K and ColE2

We next examined if ColE2 and R6K were orthogonal origins of replication. Plasmids bearing either an R6K or ColE2 origin of replication were transformed or cotransformed into cells expressing *pir, repA*, or both. We observed colonies only when a plasmid was transformed into cells possessing the cognate replication factor, as expected.

### Construction of DIAL Strains

To generate cells expressing diverse levels of the *trans*-acting factors Pir and RepA, we integrated expression cassettes with randomized ribosome binding sites (RBSs) into the genome, thereby creating strain sets JTK160 and JTK164 for *pir *and *repA*, respectively. We subsequently visualized copy number variation by transforming the libraries with reporter plasmids constitutively expressing sfGFP [20] (pBjk2741-jtk2828 or pBjk2807-jtk2828). After a preliminary fluorescence analysis of 380 clones of each type (data not shown), 24 were selected for further investigation. After a second round of fluorescence measurement, 10 variants of each type that spanned the range of observed fluorescence levels were chosen for full characterization. The sequences of the selected RBSs are reported in table 1.

**Table 1:** RBSs of *repA *and *pir *expression variants

Strain	Variant	Sequence
JTK160 (*repA*)	A	TCTAGAATACCTGATG
	B	GTGAGAAGTGAACGTG
	C	CCGAGAACGTAGGATG
	D	GGCAGAAAGTTGAATG
	E	GTGAGAAAGCTCTGTG
	F	CTCAGAACCGAATATG
	G	GTGAGAATGCTTTATG
	H	TTGAGAAAAACAGATG
	I	GGGAGAAAGATGGGTG
	J	GGGAGAAAACAAAATG

JTK164 (*pir*)	A	GCTGGAACAGGTGGTG
	B	TAAGGAATTAGGTGTG
	C	GGGGGAAGGGCATGTG
	D	TTGGGAACAATTCATG
	E	TCCGGAAGACTAGGTG
	F	GTCGGAAAGGGCTGTG
	G	GTGGGAATAGAATATG
	H	ACGGGAATGTAACGTG
	I	ACTGGAACTGCATGTG
	J	ATCGGAAACATAGGTG

Start codons are underlined.

### Characterization of DIAL Strains: Copy Number, Cell-to-Cell Variability, and Stability

**Figure 1:**
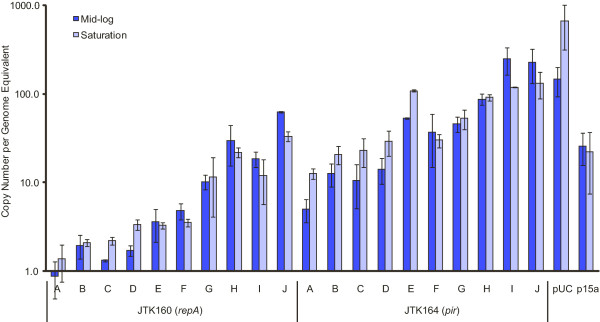
**qPCR estimation of plasmid copy number**. JTK160 (*repA*) and JTK164 (*pir*) variants were transformed with pBjk2992-jtk2541 and pBjk2993-jtk2541, respectively. JTK160J and JTK164J were also transformed with pBjo1601A-jtk2541 (p15a origin) and pBjk3057-jtk2541 (pUC origin). Two biological replicates of each sample were prepared and analyzed using qPCR. Error bars represent standard deviation.

We characterized three important properties of plasmids in the DIAL strains: copy number, cell-to-cell variation, and stability. To estimate the copy number supported by each strain, we employed qPCR to examine plasmid content both at mid log and at stationary phase (Figure 1). Based on this analysis, a ColE2 plasmid in the DIAL strains spans the range of ~1-60 copies per genome equivalent, while an R6K plasmid in the DIAL strains spans the range of ~5-250 copies per genome equivalent. This covers nearly the entire range of reported plasmid copy numbers, from single copy to nearly pUC levels. We observed that the pUC plasmid exhibited 4-5 fold increased copy numbers at stationary phase, while the p15a, R6K, and ColE2 plasmids showed ~2-fold or lower changes.

**Figure 2:**
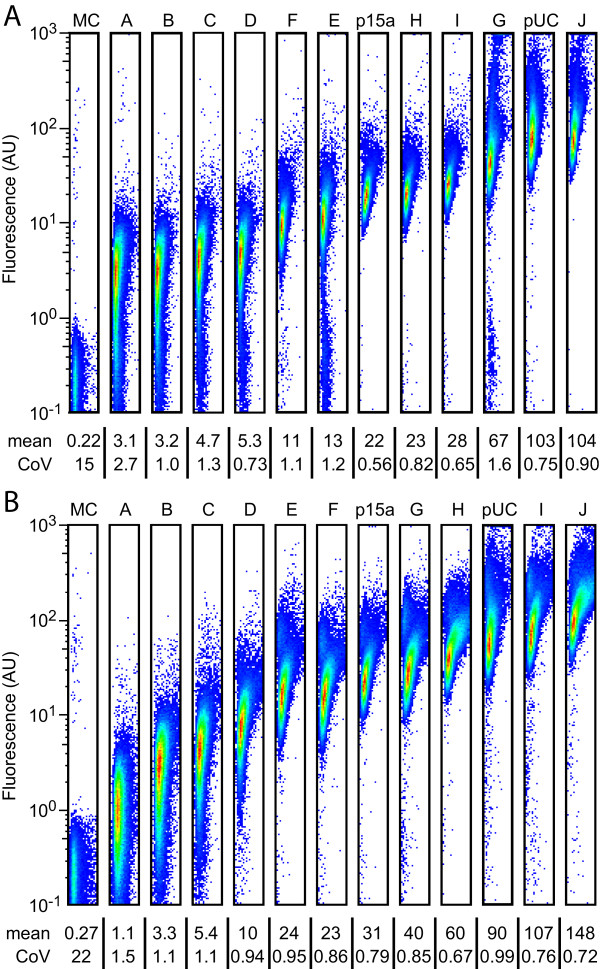
**Flow cytometric analysis of cell-to-cell variation**. JTK160 strains expressing *repA *(A) or JTK164 cells expressing *pir *(B) bearing GFP expressing plasmids (pBjk2741-jtk3026 or pBjk2807-jtk3026, respectively) were analyzed by flow cytometry. MC1061 alone or bearing p15a (pBjo1601A-jtk3026) or pUC (pBca9145-jtk3026) plasmids expressing GFP were also analyzed. Data is presented as forward scatter versus GFP fluorescence, with the mean and coefficient of variance listed below.

We next examined sfGFP expression in samples of each of the cells by flow cytometry (Figure 2) to determine cell-to-cell variability. The ColE2 and R6K plasmids generally exhibit similar distributions to p15a and pUC origins. Strains JTK160I and JTK164E, which have mean expression levels within 25% of p15a levels, have coefficients of variance that fall within 25% of p15a levels. Similarly, JTK160J and JTK164I, which have mean expression levels within 25% of pUC levels, have coefficients of variance that fall within 25% of pUC levels. It is unclear if the relatively high coefficients of variance for JTK160A and JTK164A are a result of noise at low fluorescence (as evidenced by the very high MC1061 coefficients of variance) or due to true variance in plasmid copy number or GFP expression.

**Figure 3:**
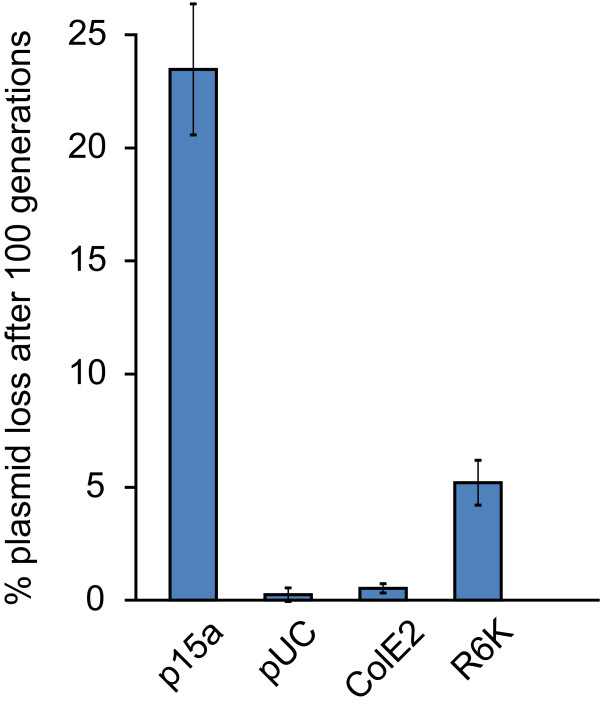
**Stability of plasmids in DIAL strains**. JTK165EI (*pir repA*) bearing pBjo1601A-jtk2828 (p15a origin), pBjk3057-jtk2828 (pUC origin), pBjk2741-jtk2828 (ColE2 origin), or pBjk2807-jtk2828 (R6K origin) was serially propagated for 100 generations and then analysed for plasmid loss. Four biological replicates of each plasmid were analysed, and error bars represent standard deviation.

Finally, we monitored the stability of plasmids in mid-level expression variants of both *pir *and *repA *after 100 generations without selection (Figure 3). Both *pir *and *repA *variants exhibited high stability, losing the plasmid in only 5.2% or 0.5% of cells, respectively. These numbers fall between the stability of the control p15a and pUC plasmids, which lost the plasmid in 23.5% or .25% of cells, respectively.

### Optimization of Violacein Expression

**Figure 4:**
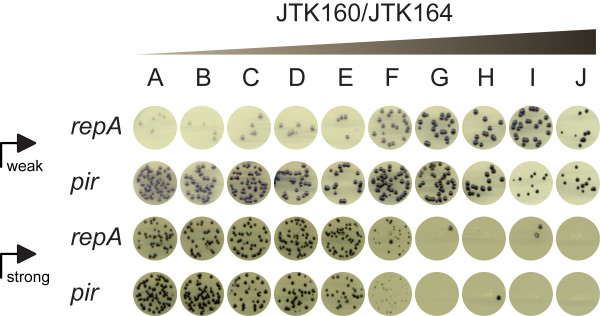
**Optimization of violacein production**. Variants of JTK160 (*repA*) or JTK164 (*pir*) were transformed with plasmids containing either a weak promoter (pBjk2741-jtk3070 or pBjk2807-jtk3070) or strong promoter (pBjk2741-jtk3080 or pBjk2807-jtk3080) driving expression of the violacein synthesis pathway. Transformants were plated as 5 μl spots. Small circles for a given strain set/plasmid combination are cropped from the same original plate image taken on a flatbed scanner. Gradient indicates the trend of copy number in strain variants.

As a simple demonstration of the utility of the DIAL strains, we optimized the expression level of the violacein biosynthesis operon, VioABCDE. While moderate production levels of the deeply purple metabolite are tolerated in *E. coli*, high levels can be toxic or cause instability [17]. We cloned the operon behind both a weak and a strong constitutive promoter to illustrate the flexibility afforded by controlling copy number. Figure 4 shows the colonies that result from transforming both weakly and strongly expressed operons (jtk3070 and jtk3080, respectively) into all of the DIAL strains. Production of violacein, as indicated by purple coloration, clearly increases as copy number increases, up to some threshold level. Beyond that threshold, colonies begin to grow at reduced rates or not at all. The large colonies in the high copy strains (JTK160G, JTK160I, and JTK164H) with a strong promoter were sequenced and confirmed to be escape mutants in which a fragment of the strong constitutive promoter has recombined out, demonstrating the risk of overstressing the cells.

## Conclusions

We have developed and characterized two sets of strains that support the R6K and ColE2 origin of replication at a wide range of different copy numbers enabling rapid exploration of gene and circuit dosage. To accomplish this, we placed the *trans*-acting replication factors from each replicon under artificial transcriptional regulation in the genome, leaving only the origins of replication on the plasmids themselves. Although negative feedback relying on elements 5' of the trans-acting factor open reading frame has been implicated as one factor helping to maintain stable copy numbers in both ColE2 [21] and R6K [14], we found that engineered cells stably maintained plasmid copy numbers despite removal of all 5' regulatory elements. This is consistent with the existence of additional feedback mechanisms, as has been suggested for both R6K [22] and ColE2 [23].

To generate copy number diversity in the DIAL strains, we created a library of RBS variants of the *trans*-acting replication factor in the genome. Although other strategies, such as the use of an inducible promoter or a library of promoters, could also have achieved diverse levels of *trans*-acting factor expression, varying the RBS enables compatibility with genetic circuits employing any promoter and maintains a consistent noise profile across strains due to stochastic transcription effects [24]. We employed a novel mechanism of generating RBS libraries in the genome: lambda red based integration of Splicing by Overlap Extension (SOEing) [25] PCR products. Multiplex automated genome engineering [26] has also been employed for creating libraries of modified genomic RBSs. While that process is a powerful method for modifying genes already in the genome, this work required simultaneous modification and introduction into the genome of an exogenous gene. In such cases, PCR based integration is an excellent option for library construction, particularly where a relatively small number of variants (<10,000) is sufficient to isolate the desired functionality.

The DIAL strains are the first tool capable of systematically varying genetic circuit dosage without altering the local genetic context. Previous studies examining the impact of circuit dosage in prokaryotes have been largely theoretical (e.g. [27,28]), and in eukaryotes focus only on low (~1-2) copy numbers (e.g. [29]). Because the theoretical predictions suggest that circuit dosage has a significant impact on the function of some genetic circuits, it is important to empirically verify the robustness or fragility of different circuit architectures. Using the DIAL strains, network behaviour and expression noise can be rapidly assessed at a wide variety of different circuit dosages.

Of great practical use, the DIAL strains offer a rapid, facile mechanism for determining desired expression levels, making it a tool with broad applicability in genetic engineering. The trivial operation of transforming the same plasmid into different strains is sufficient to provide information on the maximum tolerated expression level for a given protein, pathway, or circuit, and screening of viable colonies reveals the optimal expression level for a desired phenotype. We demonstrated this simple capability by optimizing expression of the violacein biosynthesis pathway, which in excess produces moderate toxicity in *E. coli*. Regardless of whether that starting point was a weakly or a strongly expressed operon, deeply purple yet healthy cells were isolated when matched with the appropriate strain. Knowing the optimal gene dosage can be leveraged to change the context of a gene or operon without altering the phenotype. Since the copy number is known, any change in protein dosage resulting from changing the context of the system (such as by integration into the genome) can be compensated for by using existing tools such as the RBS calculator [7] or a set of standard promoters [5].

Importantly, the ColE2 and R6K origins are orthogonal and can co-exist in the same cell, and the two sets of DIAL strains were designed to enable ready combination of both *trans *factors into a single strain by P1 transduction. Having a single set of cells with both orthogonal origins allows both the relative and absolute levels of two genes or sets of genes to be optimized by, for example, co-transformation into a pool of competent cells. Although R6K has already seen widespread use because of its ability to split into *trans *and *cis *elements, having a variety of copy number variants available for both R6K and ColE2 provides an even more powerful toolset for expression level optimization and balancing, circuit dosage investigation, and novel selection schemes.

## Methods

### Media

Strains were propagated in LB broth and LB agar plates, with addition of 100 μg/ml ampicillin sodium salt, 50 μg/ml spectinomycin dihydrochloride pentahydrate, 25 μg/ml kanamycin sulphate, and/or 10 μg/ml trimethoprim if appropriate.

### Plasmids

Plasmids were constructed using BglBrick standard assembly [30]. Full sequences of plasmids are available in Additional file 1, Table S1. The replicon of ColE2-P9 [16] is referred to as ColE2 for convenience. The gamma origin of R6K is similarly referred to simply as the R6K origin.

### Genomic RBS Library Construction

**Figure 5:**
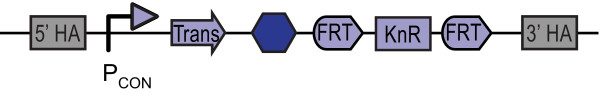
**Schematic of templates constructed for genomic integration**. Plasmids pBjk2648r-jtk2951 and pBjk2741-jtk3041 both contain: 1) a ~500 bp homology arm (HA) matching the 5' end of the genomic insertion site 2) a constitutive promoter (P_CON_) 3) the ORF of the trans-acting replication factor (Trans) 4) a terminator 5) a kanamycin resistance cassette (KnR) flanked by FRT sites and 6) a ~500 bp HA matching the 3' end of the genomic insertion site. Full sequences can be found in Additional file 1, Table S1.

Template plasmids pBjk2648r-jtk2951 and pBjk2741-jtk3041, illustrated schematically in Figure 5, were first constructed. Splicing by overlap extension SOEing PCR [25] with degenerate oligos (table 2) was used to generate RBS variants (NNNGGAANNNNNNRTG for *pir *and NNNAGAANNNNNNRTG for *repA*) of the cassettes on the template plasmids. The final PCR products were gel purified using Zymo columns according to the manufacturer's instructions, and then used to modify the genome of strain MC1061 [31] by the procedure of Datsenko and Wanner [32]. The resulting libraries consisted of 10,000 members each, and were pooled before preliminary transformation with fluorescent protein expressing plasmids and analysis of fluorescence levels. Variants ultimately selected for full characterization were P1 transduced into MC1061 cells before further analysis to eliminate the initial fluorescent plasmid.

**Table 2:** Oligos used for library construction


*pir*	pBjk2741-jtk3041	5'-3' oligos
	Outer oligo F	CACATGGCGACCAGATCAATAC
	Outer oligo R	ATTGCCGCAGGTGGAAAC
	Inner oligo F	GGTACAGTGCTAGCGGATCTNNNGGAANNNNNNRTGAGACTCAAGGTCATGATGG
	Inner oligo R	GATCCGCTAGCACTGTACC

*repA*	pBjk2648r-jtk2951	5'-3' oligos
	Outer oligo F	GGATTTTCCTTGTTTCCAGAG
	Outer oligo R	GCTTACGGCTTTATATTACGGG
	Inner oligo F	CTCGTCAGTAACGAGCCCTNNNAGAANNNNNNRTGAGCGCCGTACTTC
	Inner oligo R	AGGGCTCGTTACTGACGAG

### Plasmid Copy Number Estimation by qPCR

Plasmid copy number per genome equivalent was estimated using the relative quantitation method described previously [33]. Briefly, cells were subcultured 1:100 into fresh media and grown until mid-log or stationary phase before total DNA isolation using QIAamp DNA Mini kits (Qiagen) according to the manufacturer's instructions. DNA samples and 10-fold serial dilutions of a purified calibrator plasmid bearing a single copy of both *bla *and *dxs *(pBca9145-jtk3068) were then amplified on an iCycler with iQ5 real-time PCR detection system (Biorad) using previously validated primer pairs [33] for both *bla *and *dxs *(bla F: 5'-CTACGATACGGGAGGGCTTA-3' blaR: 5'-ATAAATCTGGAGCCGGTGAG-3' dxsF: 5'-CGAGAAACTGGCGATCCTTA-3' dxsR: 5'-CTTCATCAAGCGGTTTCACA-3'). Each reaction contained 25 μl: 12.5 μl Absolute QPCR SYBR Green Fluorescein Mix (Thermo Scientific), 1.25 μl each primer (10 μM), 3.75 μl H_2_O, and 5 μl sample DNA. Reaction conditions were as follows: an initial denaturation at 95°C for 10 minutes, followed by 40 cycles of 95°C for 10 seconds, 63°C for 15 seconds, and 72°C for 15 seconds. Measurements were taken at the end of each extension step. Copy numbers were calculated by using the calibrator standard curves to determine the quantity of plasmid (*bla*) and genome (*dxs*) dna for a given sample in arbitrary units, and then calculating their ratio.

### Flow Cytometry

Cells grown to stationary phage were subcultured 1:100 in fresh media, grown until mid-log, resuspended in PBS, and then examined on a Coulter Epics Xl-MCl instrument with a 488 nm excitation wavelength and 525 nm emission bandpass filter.

### Stability Analysis

Single colonies were picked and grown to stationary phase under selection. Cells were then subcultured 1:10^6 ^and grown back to stationary phase without selection, which corresponds to 20 generations of growth. The dilution and regrowth was repeated serially for 100 generations, at which point dilutions of cells were plated on non-selective media, and colonies were examined for sfGFP fluorescence as an indicator of plasmid presence.

## Competing interests

The authors declare that they have no competing interests.

## Authors' contributions

JTK and JCA designed experiments. JTK and SC carried out experiments. JTK and JCA drafted the manuscript. All authors read and approved the final manuscript.

## Supplementary Material

Additional file 1 **Lists the full sequences of plasmids and strain modifications used in this study**. Click here for file
